# The impact of digital navigation on travel behaviour

**DOI:** 10.14324/111.444/ucloe.000034

**Published:** 2022-04-05

**Authors:** David Metz

**Affiliations:** 1 Centre for Transport Studies, University College London, Gower Street, London WC1E 6BT, UK

**Keywords:** travel behaviour, navigation, road traffic, minor roads, journey time, road traffic congestion

## Abstract

Digital navigation – the combined use of satellite positioning, digital mapping and route guidance – is in wide use for road travel yet its impact is little understood. Evidence is emerging of significant changes in use of the road network, including diversion of local trips to take advantage of new capacity on strategic roads, and increased use of minor roads. These have problematic implications for investment decisions and for the management of the network. However, the ability of digital navigation to predict estimated time of arrival under expected traffic conditions is a welcome means of mitigating journey time uncertainty, which is one of the undesirable consequences of road traffic congestion. There is very little available information about the impact of digital navigation on travel behaviour, a situation that needs to be remedied to enhance the efficiency of road network operation.

## Introduction

Road traffic is a major source of environmental detriment, in particular, carbon emissions and urban air pollution. Building new road capacity adds to traffic, embeds the carbon in cement and steel, as well as concreting over the countryside. There is therefore much interest in alternative means to achieve the access made possible by physical mobility, in particular using digital technologies for virtual access, as has happened widely during the coronavirus pandemic. Digital technologies are also of interest as a means to improve the effectiveness and efficiency of the transport system, for instance, digital signalling on the railway that allows trains to operate safely with shorter distances between them, thus increasing the capacity of the physical infrastructure.

Recent advances in a number of digital technologies in combination are having a significant impact on travel behaviour on the road network by providing route guidance that takes account of traffic conditions. What may be termed ‘digital navigation’ involves the use of satellite navigation system (GPS, satnav) to provide both spatial positioning to a high precision, as well as the ability to infer vehicle speeds and hence the location of traffic congestion; digital mapping; and routing algorithms to optimise journeys.

Digital mapping has essentially digitised traditional paper maps, in the same way that digital documents have digitised their paper versions. Digital maps have the capacity to accommodate vast amounts of data, including from satellite images and street level information, data that can be readily updated. The combination of satellite-based location and digital mapping provides a navigation service. For shipping and aircraft, this takes the form of a recommended course between waypoints. For road vehicles, turn-by-turn route guidance is provided.

GPS location allows the progress of vehicles to be monitored and congested conditions to be detected. Real time congestion is included in the most useful route guidance offerings, an approach pioneered by Waze, originally an Israeli tech start-up. This uses smartphones to collect crowd-sourced data about traffic conditions from users, including slow going congested traffic, and feeds back suggestions for routes with the least travel time, taking account of traffic conditions. Also provided are estimated journey times in advance of setting out. In 2013 Waze became a subsidiary of Google, which employs the Waze technology for route guidance for Google Maps. These smartphone apps are free to use, the cost of the service funded by retailers who pay to have the map show their locations. Other providers of route guidance technology that take account of traffic conditions and estimate time of arrival include TomTom and Garmin, both of which operate via devices that are either fitted to the car or are stand-alone.

While digital navigation is in widespread use by road users, remarkably little information is publicly available about performance, in particular, how routes are optimised, the suitability of recommended routes, the accuracy of estimated journey times and the impact on the functioning of the road network as a whole. Digital navigation is a technology that is changing how roads are used, yet its implications are little understood.

Existing literature is sparse. Thus, a search of the TRID transport data basis using the term ‘Waze’ yielded 46 citations, only one of which is relevant to the subject of this paper (and which is accordingly cited later in the paper). It is less useful to attempt to review past published work on this topic, to identify a specific academic knowledge gap. Rather, the approach of this paper is to outline what is known in a structured manner, to identify matters of concern, and to suggest how digital navigation may be used to improve the experience of road users generally. The examples are mainly taken from experience in the UK, in respect of which published traffic data are relatively extensive.

The main topics discussed below are: the contribution of digital navigation to the growth of traffic on minor roads; diversion of local traffic to new capacity on major strategic routes; how digital navigation can mitigate the experience of road traffic congestion; and the opportunities for optimisation of use of the road network. It will be argued that there is considerable scope for the better management and regulation of the technology to improve the functioning of road networks, provide better choices for road users, and achieve improved environmental outcomes.

## Impact of digital navigation on use of minor roads

Recent revisions to British road traffic statistics appear to show that there has been a substantial growth of motor vehicle traffic on minor roads in recent years, from 108 to 136 billion vehicle miles (bvm) between 2010 and 2019, an increase of 26%. Traffic on major roads rose from 197 to 221 bvm over the same period, an increase of 12% [1].

Road traffic statistics are based on a combination of automatic and manual traffic counts. Major roads are well covered in that traffic in all links is counted on typical days, although not every link in every year. Given the vast number of minor roads, however, it is only possible to count traffic at a representative sample of locations every year, and the observed growth is applied to minor road traffic overall. Estimates from a fixed sample may drift over time such that the sample becomes less representative of the changing minor road network. To account for any errors incurred in the fixed sample, the sample is revised every decade through a benchmarking exercise involving a much larger sample of locations.

The most recent minor roads benchmarking exercise was published in 2020, based on 10,000 representative locations [2]. Overall, the benchmark adjustment for 2010–2019 was 1.19, which is the factor to be applied to 2019 data set from the original sample locations to bring this to the observed traffic level based on the benchmark sample. There is significant regional variation in the adjustment factor, from 1.35 for Yorkshire to 1.09 for the East of England, as shown in Table 1.

**Table 1.** Estimated traffic (bvm) for 2019 annual and benchmark samples

Region/country	2019 Fixed annual sample estimate (bvm) [A]	Benchmark traffic estimate (bvm) [B]	Adjustment to benchmark [B]/[A]
North East	4.8	5.8	1.20
North West	11.8	15.4	1.31
Yorkshire and the Humber	9.6	12.9	1.35
East Midlands	9.1	10.7	1.18
West Midlands	10.7	13.2	1.23
East of England	14.1	15.3	1.09
London	7.9	10.4	1.32
South East	17.4	19.7	1.14
South West	12.3	14.5	1.18
England	97.7	118.0	1.21
Scotland	10.1	10.1	1.23
Wales	6.3	7.8	1.00
Great Britain	114.1	135.8	1.19

Source: DfT [2], figure 3.

Within the class of minor roads, for B roads nationally the adjustment factor is 1.25 and for the smaller C roads it is 1.17; while for urban roads it is 1.22 and for rural roads it is 1.15. Applying the regional weightings yields an increase in traffic on minor roads overall of 26%, as noted above, whereas the increase based on the original sample would have been 6%. The previous benchmarking exercise published in 2009 found a smaller overall adjustment factor of 0.95, with a regional range of 0.81–1.08 [3].

**Figure 1:**
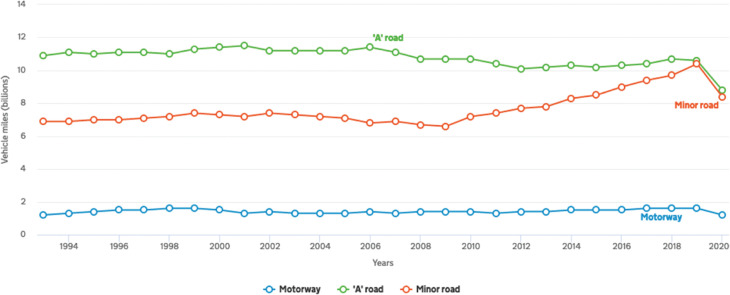
Traffic in London by road type. Source: Department for Transport, Road Traffic Statistics.

Data for minor roads’ traffic for intermediate years between benchmarks have been adjusted pro rata, to avoid a step change in the reported traffic data. This is illustrated in Figure 1 for London, which shows a steady increase in traffic on minor roads from 2010, prior to the drop arising from the pandemic.

The substantially greater adjustment required following the recent benchmarking, compared with the earlier exercise, suggests that there has been a significant real change in the use of minor roads, beyond errors arising from drift in the sample. Importantly, had the increase in minor road use been even across the country, the traffic estimation based on the sample would have been close to that from the benchmark exercise. Hence, the major difference between the sample and the benchmark suggests considerable heterogeneity of minor road traffic growth.

The UK Department for Transport (DfT) has created a revised minor roads representative sample of 4400 locations from the latest benchmark data, which will be used for the coming decade. However, the basis for creation of the new sample has not been stated. A comparative analysis of the previous and the new samples might have yielded insight into the changes that have been happening to traffic on the minor road network, to aid the selection of a new sample. The representative nature of the new sample must therefore be in question if the reasons for the failure of the previous sample to reflect reality are not understood and addressed (considered further below).

Transport for London (TfL), responsible for public transport and major roads in the city, has recognised this uncertainty. TfL’s annual report on travel in London discussed the implications of the minor roads traffic correction [4]. The revisions mean that, for 2018, the DfT estimate of vehicle kilometres for London was 20% higher than the number previously reported for that year by TfL. TfL’s previous estimate had suggested a fall of 1.8% in vehicle kilometres in London between 2009 and 2018, whereas the revised series suggested an increase of 17.9% over the same time period, this change wholly arising from revisions to the minor road estimates. TfL noted that it was working through how the DfT had made their assessment, and what this could mean for London data sets. The inconsistency with TfL’s data has prompted the DfT to review minor road traffic methodology [5].

One factor contributing to the growth of traffic on minor roads is the increase in van traffic, including that arising from the growth of online shopping with home deliveries. The number of vans (light commercial vehicles) registered in Britain increased by 28% between 2010 and 2019 [6]. Total van traffic increased by 34% over this period, with an increase of 49% on urban minor roads compared with 10% on urban ‘A’ roads, although ‘delivery/collection of goods’ was less important in respect of journey purpose than ‘carrying equipment, tools or materials’ [7]. However, in 2019 van traffic amounted to 15% of traffic on urban minor roads, and 19% on rural minor roads, cars being responsible for 82% and 78% of traffic, respectively. Thus, the growth of van traffic on minor roads could be responsible for only a small part of the overall traffic growth on these roads.

A possible cause of the changed distribution of traffic on minor roads arises from intentional interventions aimed at reducing such traffic. It has long been the practice to discourage ‘rat running’ on urban minor roads by means of suitable physical control measures. The creation of street environments supporting a shift from private car use to active travel – walking and cycling – has been an aim of recent policy initiatives to create low-traffic neighbourhoods [8]. In response to the COVID pandemic, the DfT issued statutory guidance that local authorities in areas with high levels of public transport use should take measures to reallocate road space to walking and cycling, to reduce crowding on buses and trains [9]. Such measures would lessen traffic in certain locations while possibly increasing it in others through diversion. However, the net effect of intentional interventions would be to reduce traffic overall, so this cannot account for the reported growth of traffic on minor roads.

Accordingly, the most likely main contribution to the large growth of traffic on minor roads is the widespread use of digital navigation, which makes possible the general use of minor roads that previously were largely confined to those road users with local knowledge, as well as extending such local knowledge. Diversion to minor roads is likely to occur when major roads are congested and adjacent minor roads offer an alternative; this represents an effective increase in the capacity of the road network, so generating additional traffic – an example of induced traffic, discussed below. The heterogeneity of growth of minor roads traffic, noted above, is consistent with diversion prompted by major road congestion, as minor roads located far from major roads would not be affected by such diversions. It would be desirable to investigate the past growth of traffic on minor roads as a function of congestion on adjacent major roads and the feasibility of diversion to minor roads, to inform the selection of a representative sample of minor roads for future traffic monitoring.

## Impact of digital navigation on use of major roads

As well as encouraging use of minor roads, digital navigation may divert traffic from local roads to roads intended for longer distance traffic (motorways and major roads of the UK Strategic Road Network, other nomenclature used for similar roads elsewhere). One case where such a diversion may have occurred is the widening of the M25, the London orbital motorway, between junctions 23 and 27 to the north of the city, for which detailed traffic monitoring was carried out before and for 3 years after widening [10]. The addition of an additional lane in each direction led to a substantial increase in traffic, well above the forecast made using a regional traffic model, such that there was no increase in traffic speed beyond the first year after opening. The model projected travel time saving benefits for both business and non-business users, but for the latter these were almost entirely offset in economic value by increased vehicle operating costs, reflecting diversion of local trips to take advantage of the time saved by using the motorway. The nature of the additional traffic, over and above that projected by the model, cannot be deduced from traffic counts, but it is likely that much, perhaps most, arises from more local journeys diverting. The projected benefit–cost ratio (BCR) based on the modelling was 2.9, which ranked as high value for money, but the outturn was far lower.

**Figure 2:**
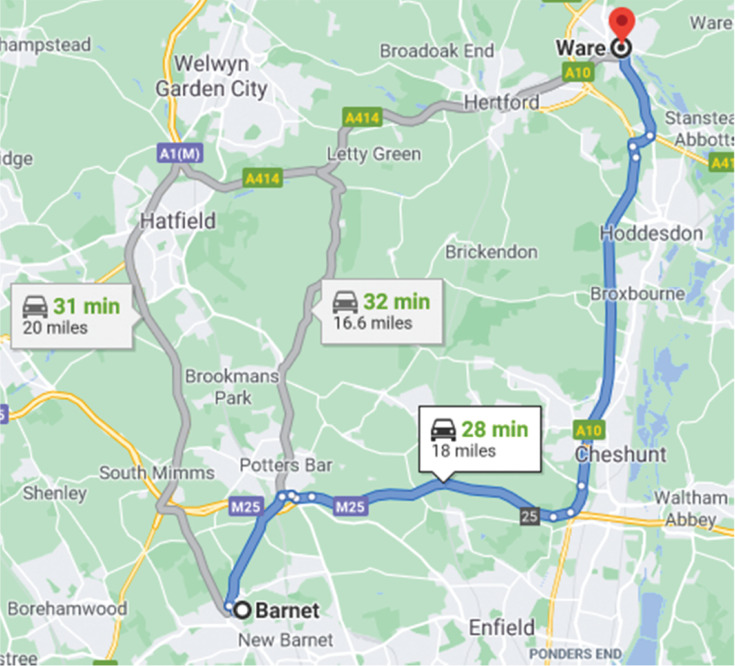
Google Maps screenshot, weekday mid-morning.

The contribution of digital navigation in facilitating such a diversion cannot be inferred from the available data, but it is plausible as illustrated by the example in Figure 2, which shows a journey from Barnet in North London to Ware in Hertfordshire, a distance of about 16 miles on the most direct route via lesser roads. However, the fastest route involves a diversion to the M25 for a saving of 4 min in this case. Regular users of digital navigation would have up-to-date information for each journey.

The M25 case study suggests that digital navigation may increase the likelihood that local traffic would take advantage of the capacity increase of major routes in the vicinity of urban areas that generate a lot of traffic. These are the locations where the Strategic Road Network is under greatest stress and where investments to increase capacity are thought to be most needed. Yet this diverted local traffic negates the benefits expected for long-distance road users and so undermines the economic case for the investment.

## Mitigating the impact of road traffic congestion

The development of digital navigation offers probably the best means available to mitigate the impact of road traffic congestion. Congestion arises in or near areas of high population density and high car ownership, where the capacity of the road network is insufficient to cope with all the trips that might be made. Drivers are deterred by the prospect of time delays and so make other decisions – to travel at a different time, by a different route, by a different mode, to a different destination (where there are options, as for shopping), or not to travel at all (by shopping online, for instance). Congestion is therefore substantially self-regulating, in that if traffic increases, delays worsen, and more potential users are deterred on account of the time constraint [11].

Adding road capacity to a congested route initially reduces delays, which attracts some of the previously deterred drivers, so that more car journeys are made, but the resulting additional traffic restores congestion to what it had been. This additional traffic arising from increasing road capacity is known as ‘induced traffic’ and is the consequence of road users taking the benefit of investment that permits faster travel by travelling further to gain access to more destinations, opportunities and choices (for a recent discussion see [12]). Induced traffic is the basis of the maxim that we cannot build our way out of congestion, which we know from experience to be generally true – for instance, why the successive addition of extra lanes to the M25 that orbits London failed to offer more than short-term relief of congestion.

Digital navigation that takes account of congestion in real time can offer less congested routes, thus making better use of the existing road network and reducing road users’ exposure to congestion. One problem that may arise is that traffic may be diverted to unsuitable roads, where local environments and neighbourhoods may be adversely affected, or even where large vehicles can become obstructed. Some satnav systems are designed specifically for heavy goods vehicles and avoid use of unsuitable routes, taking account of size, weight and load of the vehicle. However, it is not clear how many truck drivers use a bespoke system, which can be expensive, and how many rely on more popular satnav devices designed for cars or free-to-use options on a smartphone. Diversion to unsuitable routes is a problem that could be mitigated through collaboration between digital navigation providers and road authorities (discussed later).

Beyond the rerouting of traffic to less congested roads, there is a feature of digital navigation that mitigates the unwelcome experience of traffic congestion – the prediction of journey time, or estimated time of arrival (ETA). Anecdotal evidence suggests that for road users the uncertainty of journey time is a more important adverse consequence of congestion than lower speed. One survey of road users in England asked about priorities for increased expenditure on motorways: almost half of respondents ranked improved flow and reduced congestion as a priority, compared with less than a quarter wanting reduced journey times [13]. Accordingly, it seems likely that an important benefit of digital navigation is the forecast of ETA in the light of prevailing traffic conditions on the selected route, in this way substantially reducing journey time uncertainty. Regrettably, there is a lack of publicly available data on performance. However, a recent study reported substantial improvements in the accuracy of ETA prediction by Google Maps through the application by DeepMind of Graph Neural Networks, which allowed performance to be improved by machine learning [14].

## Optimising road networks

While diversion onto less congested routes may be helpful for users of digital navigation, there is a question as to whether this is optimal for users of the road network as a whole. Digital navigation employs proprietary algorithms whose performance is difficult to assess externally. An algorithm might respond to build up of congestion by diverting all traffic to a single alternative route until that became congested, repeating the process to spread traffic across available routes until congestion abated. Or the algorithm might spread traffic across all available routes at the outset. And the algorithm might anticipate the build-up of congestion based on historic experience. But in any event, the routing algorithm used by one provider would not take account of the activities of another provider. There is little published information on the design and performance of route guidance devices. Reports in the informal literature suggest that Dijkstra’s shortest-path algorithm is generally used in navigation systems. A rare, detailed paper, whose authors include Microsoft staff, describes a method for predicting the probability distribution of travel time for specified routes at chosen times, which is reported to perform well on the Seattle road network [15].

Estimation of journey times before setting out may increase the operational efficiency of the road network. To a first approximation, there are two kinds of road users. Those who need to be at their destination at a particular time, whether to get to work or a meeting or to deliver time-critical goods, are able to use predictive journey time information to decide when best to set out. In contrast, there are those who are more flexible in timing their trips, for instance, for shopping, leisure activities or delivering goods in wide time slots; this group could use estimated journey times to minimise their exposure to congested traffic. This is a potentially win–win situation, in that the more the flexible road users can avoid peak traffic, the less traffic at peak times experienced by those not flexible.

When only a few people used digital navigation that took account of congestion, there were benefits for those diverted on to less congested routes as well as for those that did not divert, who experienced rather less traffic. But as more drivers use route guidance as it exists, and as diversions become more common, the net benefit to society is less clear. Nevertheless, there is potential for digital navigation technologies to make a significant impact on how the road network functions, with implications for users beyond those equipped with the technology – a reason for collaboration between providers of navigation services and road authorities to achieve the best outcomes for all road users and for those exposed to traffic in their local environment. There is therefore a case for a regulatory regime to govern the operations of digital navigation providers.

The road system is well regulated to achieve safety and efficiency. Vehicles must be certified as safe and commonly older vehicles are tested annually; drivers must be licenced and are penalised for traffic offences; and the road infrastructure is built to prescribed standards. Given the potential scale of impact of digital navigation devices on network operations, arguably a licensing regime would be appropriate for providers. This might require information to be exchanged with road authorities, guidance to be accepted to avoid adverse environmental and social impacts, and mutual collaboration to optimise the operational efficiency of the network as a whole.

In Britain, there is in fact legislation in existence, the Road Traffic (Driver Licensing and Information Systems) Act 1989, which requires dynamic route guidance systems that take account of traffic conditions to be licenced by the government. The licence could include conditions concerning the roads that should not be included in route guidance and provision to road authorities of data on traffic conditions. The intention of the legislation was to facilitate the introduction of a pilot route guidance system that had been developed by the government’s Transport and Road Research Laboratory, although in the event this was not taken forward. In practice this legislation has been disregarded as no licences have been issued.

Examples of voluntary data sharing exist. In London, the transport authority, TfL, makes data freely available to app developers and is a partner with Waze’s Connected Citizens Programme, which provides cities with real time data on traffic disruptions [16]. Uber, the ride-hailing provider, also provides anonymised data on traffic movement for many cities, based on data from its fleet of vehicles. Much is known about the impact of ride-hailing on traffic in cities in the United States (US) because performance data can be extracted from the application programme interfaces of the operators [17], which has prompted the companies to volunteer data provision to help cities address urban transportation needs.

Such sources of data are of benefit to city authorities that operate urban traffic management systems aimed at optimising traffic flows by varying the timing of traffic signals. In London, where three quarters of congestion is the result of excess demand and one quarter from planned events or unplanned incidents, 75% of the 6000 sets of traffic signals vary their timing continuously to optimise flow across both individual junctions and the network as a whole, reducing delays at junctions by about 13%. The benefits from such dynamic traffic signal technology were shown during the 2012 London Olympic Games when major changes in flow were managed successfully [18].

A related question concerns the scope for improving traffic flows by means of the algorithms for digital navigation used by individual drivers. A study using data collected before and during the 2016 Rio de Janeiro Olympics, including data from Waze, compared the expected journey times for three scenarios, according to whether drivers followed their usual routes, chose routes with the shortest time for the individual, or were directed to routes that were best for travellers as a whole. The finding of this modelling study was that adhering to usual habits involved up to 7% longer journey times during the Games compared with both the ‘selfish’ and ‘altruistic’ approaches [19]. This suggests some modest benefits for users of digital navigation, although the advantage of attempting to optimise use of the network for all users is unclear.

The European project SOCRATES^2.0^ involves a consortium of public and private organisations that is attempting to pilot smart traffic and navigation services, one of which involves route guidance to optimise network traffic flows with cooperative traffic management in the Amsterdam region. No findings are yet available [20].

## Discussion and conclusions

Although the evidence is very limited at present, it seems clear that the advent of digital navigation is significantly changing how the road system is used by offering faster journeys, particularly when roads are congested. The general effect is to increase the usable capacity of the network, thereby increasing operational efficiency. However, there are disadvantages. There is evidence of diversion of traffic onto minor roads that had not previously experienced much through traffic, with deleterious environmental consequences. And local traffic can be diverted to take advantage of additional capacity on major routes, reducing the expected benefit to long-distance road users. Arguably, the main benefit of digital navigation is to reduce journey time uncertainty, which is the consequence of road traffic congestion that most concerns roads users.

It seems likely that digital navigation could be managed to achieve better outcomes, not just for users of the devices but for all road users. This would involve collaboration between the providers of navigation services and road authorities. The aims would be to avoid the use of unsuitable roads and to optimise network performance at times of stress, whether due to daily congestion, major incidents or adverse weather conditions. This should not affect the business models of the providers, which depend either on selling direction-finding for retailers’ websites or mapping services to vehicle manufacturers. As noted above, there is a legislative framework in the UK that could be used to facilitate such collaboration.

There is a noteworthy contrast between the lack of regulation and lack of understanding of digital navigation and the position of the other important new digital technology, the digital platform as used by ride-hailing businesses to match demand to supply, as exemplified by Uber. Much is known about the impact of ride-hailing on traffic in US cities, in part because the authorities are able to require provision of data as a condition of the companies’ operating licence [21].

Although research findings in relation to digital navigation are as yet very limited, there are relevant theoretical studies of the application of game theory to the provision of routing information to travellers [22,23]. As yet, such theoretical analyses cannot be validated against empirical observations, although there are prospects for doing so given that the information generated by the navigation devices is vast, forming part of the ‘Big Data’ made available by the range of new digital technologies. Harrison et al. [24] have reviewed the extensive research literature bearing on the potential of GPS-based data sets to enhance transportation modelling. In the context of the present discussion, the research requirement relates to the dynamic functioning of the road network, for which access to the data available to the providers of digital navigation services would seem essential. The most likely route to such access would be by means of a regulatory regime.

A regulatory regime would involve the licensing of providers of digital navigation services, with conditions relating to the avoidance of unsuitable routes, provision of information to road authorities and a duty to collaborate with authorities to optimise the operations of the road network for the benefit of all users.

Such optimisation of the functioning of the road network through more effective use of digital technologies offers an alternative to costly civil engineering to increase capacity, particularly as the costs of digital navigation are not borne by public road authorities. Indeed, the economic benefits of civil engineering investment in additional road capacity are likely overstated, on account of the diversion of local traffic of little economic value to take advantage of increases in major road capacity, thus negating the economic benefit to long-distance users, as the M25 smart motorway case study discussed above illustrates. A prospective economic analysis of the future UK highways investment programme estimated that 10 planned smart motorway investments would have an average benefit–cost ratio of 2.4 [25], yet this could be very substantially overstated on account of traffic diversion facilitated by digital navigation. Accordingly, the economic case for the very substantial public expenditure on new road capacity is much weaker than is usually supposed.

More generally, the wider use of digital navigation may increase overall traffic growth by effectively increasing available road capacity. This would need to be taken into account in traffic modelling at all levels, national, regional and local. Such an increase in road traffic volumes is problematic when policy is concerned to decarbonise the transport system and encourage active travel – walking and cycling. Clearly, a far better understanding of the impact of digital navigation is needed, to optimise the operations of the road network both in the present and the future.

If it proves possible to use digital navigation to modify driver behaviour to get better outcomes for all road users, this would be an example of a ‘nudge’ – an intervention that structures the choices available to help individuals to make better choices without restricting their freedom to choose. Thaler [26], proposer of the nudge concept, cites satnav technology on smartphones as an example: you decide where you want to go, the app offers possible routes, and you are free to decline the advice if you decide to take a detour. However, for nudging to succeed, we would need to be confident that there is indeed a benefit to all road users, whether nudged or not.
